# Differential association between S100A4 levels and insulin resistance in prepubertal children and adult subjects with clinically severe obesity

**DOI:** 10.1002/osp4.381

**Published:** 2019-12-02

**Authors:** Siri D. Taxerås, María Galán, Laura Campderros, Irene Piquer‐Garcia, Silvia Pellitero, Eva Martínez, Rocío Puig, Icíar Lucena, Jordi Tarascó, Pau Moreno, José Balibrea, Joan Bel, Marta Murillo, María Martínez, Marta Ramon‐Krauel, Manel Puig‐Domingo, Francesc Villarroya, Carles Lerin, David Sánchez‐Infantes

**Affiliations:** ^1^ Department of Endocrinology and Nutrition Germans Trias i Pujol Research Institute Barcelona Spain; ^2^ Institut de Recerca del Hospital de la Santa Creu i Sant Pau‐Programa ICCC Barcelona Spain; ^3^ CIBER de Enfermedades Cardiovasculares (CIBERCV), ISCIII Madrid Spain; ^4^ Department of Biochemistry and Molecular Biology, Institute of Biomedicine University of Barcelona Barcelona Spain; ^5^ Biomedical Research Center (Red Fisiopatología de la Obesidad y Nutrición) (CIBEROBN), ISCIII Madrid Spain; ^6^ Biomedical Research Center (Red Fisiopatología de la Diabetes y enfermedades metabólicas) (CIBERDEM), ISCIII Madrid Spain; ^7^ Department of Surgery Germans Trias i Pujol Research Institute Barcelona Spain; ^8^ Metabolic and Bariatric Surgery Unit, EAC‐BS Center of Excellence Vall d'Hebron University Hospital Barcelona Spain; ^9^ Department of Pediatric Germans Trias i Pujol Research Institute Barcelona Spain; ^10^ Endocrinology Department Institut de Recerca Sant Joan de Déu Barcelona Spain; ^11^ Hospital Sant Joan de Déu Barcelona Spain

**Keywords:** insulin resistance, obesity, prepubertal children, S100A4

## Abstract

**Objectives:**

S100A4 has been recently identified as an adipokine associated with insulin resistance (IR) in adult subjects with obesity. However, no data about its levels in children with obesity and only a few approaches regarding its potential mechanism of action have been reported. To obtain a deeper understanding of the role of S100A4 in obesity, (a) S100A4 levels were measured in prepubertal children and adult subjects with and without obesity and studied the relationship with IR and (b) the effects of S100A4 in cultured human adipocytes and vascular smooth muscle cells (VSMCs) were determined.

**Methods:**

Sixty‐five children (50 with obesity, age 9.0 ±1.1 years and 15 normal weight, age 8.4 ±0.8 years) and fifty‐nine adults (43 with severe obesity, age 46 ±11 years and 16 normal weight, age 45 ±9 years) were included. Blood from children and adults and adipose tissue samples from adults were obtained and analysed. Human adipocytes and VSMC were incubated with S100A4 to evaluate their response to this adipokine.

**Results:**

Circulating S100A4 levels were increased in both children (*P* = .002) and adults (*P* < .001) with obesity compared with their normal‐weight controls. In subjects with obesity, S100A4 levels were associated with homeostatic model assessment‐insulin resistance (HOMA‐IR) in adults (*βstd* = .42, *P* = .008) but not in children (*βstd* = .12, *P* = .356). Human adipocytes were not sensitive to S100A4, while incubation with this adipokine significantly reduced inflammatory markers in VSMC.

**Conclusions:**

Our human data demonstrate that higher S100A4 levels are a marker of IR in adults with obesity but not in prepubertal children. Furthermore, the in vitro results suggest that S100A4 might exert an anti‐inflammatory effect. Further studies will be necessary to determine whether S100A4 can be a therapeutic target for obesity.

## 1 INTRODUCTION

According to updated World Health Organization (WHO) reports, overweight and obesity worldwide have almost tripled since 1975^1^. In 2016, more than 1.9 billion adults (older than 18 years) were overweight, and out of these, 650 million were obese. The problem is not only increasing in adults but also in adolescent and children (5‐18 years), where more than 340 million were either overweight or obese in 2016.^1^ Hallmarks of the obesity state, including visceral fat accumulation and low‐grade chronic inflammation, are associated with increased secretion of pro‐inflammatory cytokines and the development of metabolic diseases including cardiovascular disease and type 2 diabetes (T2D).^2^, ^3^

The pro‐inflammatory state plays an important role in insulin resistance,^2^ but the link between obesity and the development of T2D is not well‐characterized. It is known that white adipose tissue (WAT) has an endocrine role through the secretion of signalling molecules such as adipokines by adipocytes and other cell types present in adipose depots. These molecules participate in a hormonal network communicating several tissues and organs including muscle, liver, and brain.^4^ Moreover, adipose tissue expansion occurring during obesity also leads to immune cell recruitment. Therefore, adipose tissue may be considered an immunological organ since these immune cells can secrete pro‐inflammatory and anti‐inflammatory cytokines affecting adipocyte biology, metabolism, and systemic inflammation, and being a key factor in insulin resistance development and obesity‐related pathologies.^2^, ^5^ However, some patients with obesity show normal glucose homeostasis,^6^ and previously unrecognized molecules could be involved in this situation. Moreover, browning of subcutaneous WAT (appearance of thermogenic beige/brite adipocytes in WAT depots) has been associated with protection against obesity and improvement of associated metabolic alterations.^7^ Finally, angiogenesis is a key process required for adipogenesis in vivo and has an important role in inflammation‐induced adipose tissue expansion.^8^ In the normal weight state, adipose tissue is a well‐vascularized tissue.^9^ During the early stages of obesity development, angiogenic factors are overexpressed to facilitate healthy adipose tissue expansion and confer protection from metabolic insults.^10^ Therefore, a better understanding of adipose tissue endocrine, immunological and angiogenic capability, and the identification of novel potential biomarkers and therapeutic targets to prevent and treat T2D is one of the goals in obesity field.

S100A4 is a member of the S100 calcium‐binding protein family and has been reported to be involved in controlling cell growth, differentiation, survival, and migration in different cell types, as well as in energy metabolism.^11^, ^12^ S100A4 was identified by Ebradlize and colleagues as a protein associated with tumour metastasis.^13^ S100A4 binds to cytoskeletal proteins including F‐actin^14^ and nonmuscle myosin heavy chains.^15^ S100A4 gene expression is elevated in activated macrophages.^16^ A recent report has identified S100A4 as a cytokine secreted by adipose tissue mainly by precursors and immune cells, with a potential role as a marker of subcutaneous WAT dysfunction and insulin resistance in adult individuals with severe obesity.^17^ Moreover, a study performed in mice postulates that S100A4 protects against diet‐induced obesity and inflammatory state.^18^

In this study, our hypothesis was that S100A4 would be elevated and directly correlated to insulin resistance in children with obesity as well as in adults. To address this hypothesis, S100A4 levels in children and adult patients with and without obesity were quantified and their relationship with insulin resistance was determined. Moreover, the effects of S100A4 in human adipocytes and vascular smooth muscle cells (VSMCs) were studied.

## 2 MATERIALS, SUBJECTS AND METHODS

### 2.1 Children study

Data from this cohort have been previously published, reporting the effects of lifestyle intervention on the metabolomic profiles in blood and urine.^19^, ^20^ In the present study, we quantified S100A4 levels in the blood samples to evaluate potential associations with glucose homeostasis. Briefly, children attending the Obesity Unit at the Sant Joan de Déu Hospital were recruited between January 2013 and December 2014. All parents signed the informed consent form. Inclusion criteria were (a) age from 7 to 10 years of age; (b) with obesity, defined as body mass index‐standard deviation score (BMI‐SDS) greater than 2 standard deviations for a given age and sex, using the WHO standards; and (c) prepubertal, defined as Tanner stage I breast development for girls and testicular volume less than 4 mL in boys. Exclusion criteria included any form of endocrine obesity, major congenital or chronic disease, drug‐induced obesity, use of drugs for weight loss, or involvement in another weight‐loss programme. Fifty‐three children with obesity were initially enrolled in the study; plasma samples were available from 50 of these subjects at the hospital's Biobank. Fifteen prepubertal normal‐weight children (BMI‐SDS between −1 and 1 standard deviations) between 7 and 10 years of age were also recruited for the study. Plasma samples from all subjects were obtained in the morning after an overnight fast by qualified personnel at the hospital.

### 2.2 Adult subjects study

All participants gave their written informed consent before collecting clinical data. Forty‐three patients (11 males and 32 females) with severe obesity were included in this study. Ten of them were diagnosed with T2D, and eight had pre‐diabetes. All the patients were evaluated by the same endocrinologist (S. P.) following the institutional protocol for bariatric surgery between September 2012 and October 2015, according to criteria for bariatric surgery formulated in Spanish Position Statement between Obesity, Endocrinology, Diabetes and Surgery Societies.^21^ Inclusion criteria were (a) adults with BMI greater than 35 kg m^−2^ and (b) adults with BMI less than 26 kg m^−2^ and normal glucose levels (less than 100 mg dL^−1^). Exclusion criteria included any form of endocrine obesity, major congenital or chronic disease, drug‐induced obesity, use of drugs for weight loss, or involvement in another weight‐loss programme. Demographic and clinical data, including age, history of diabetes, and hypertension were prospectively recorded in all subjects. Visceral adipose tissue was obtained from a subset of these patients (n = 16). These samples were collected during bariatric surgery and frozen at −80°C until they were used. Sixteen normal‐weight healthy subjects were included as a control group to measure circulating levels of our molecule of interest. Plasma samples were collected after a 12‐hour fasting and stored at −80°C in the Biobank for the Health Sciences Research Institute Germans Trias i Pujol Foundation.

### 2.3 Circulating parameters

Glucose, insulin, and glycated haemoglobin (HbA1c) levels were measured in the certified core clinical laboratory at the hospital. The homeostatic model assessment‐insulin resistance (HOMA‐IR) was calculated.

S100A4 circulating levels were measured using a human protein S100A4 ELISA kit (Cusabio Biotech Co, LTD, China). High molecular weight (HMW) adiponectin levels were measured using a human protein HMW‐adiponectin ELISA kit (R&D Systems, China).

### 2.4 Cell culture

#### 2.4.1 Human aortic VSMCs

Human aortic VSMCs were obtained by using a modification of the explant technique and cultured as previously described.^22^ Human VSMC were obtained from nonatherosclerotic aortas of hearts removed on occasion of transplant surgery at the Hospital de la Santa Creu i Sant Pau (Barcelona, Spain) and cultured in M199 (Gibco, Carlsbad, CA, USA) supplemented with 20% fetal calf serum (FCS), 2% human serum, 2 mmol L^−1^ l‐glutamine, and antibiotics as previously described (22). Cells from four different donors between passages 3 and 6 were seeded in six‐multiwell plates. Subconfluent cells were starved in medium supplemented with 1% fetal bovine serum (FBS) for 24 hours prior to the addition of S100A4 (37.5, 75, or 150 ng mL^−1^) recombinant protein (R&D Systems) or vehicle (phosphate‐buffered saline [PBS] 1×) for 24 hours in the presence or absence of TNF‐α (50 ng mL^−1^) (Sigma Aldrich) used as positive controls of inflammatory stimuli.

#### 2.4.2 Human adipocytes

Human SGBS^23^ were used as a model of human adipocyte cells in culture. Cells were maintained with DMEM/F12 supplemented with 10% heat‐inactivated FBS, 32μM biotin and 16μM pantothenic acid. For differentiation, cells were incubated with DMEM/F12 supplemented with 32μM biotin, 16μM pantothenic acid, 25nM dexamethasone, 500μM IBMX, 2μM rosiglitazone, 10 μg mL^−1^ transferrin, 20μM insulin, 100nM cortisol, and 0.2nM T3 for 7 days. Cells were then incubated with DMEM/F12 supplemented with 32μM biotin, 16μM panthotenic acid, 10 μg mL^−1^ transferrin, 20μM insulin, 100nM cortisol, and 0.2nM T3 for seven more days or until they reach more than 90% of differentiation, where indicated cells were treated with 1mM cAMP (Sigma‐Aldrich) and S100A4 (150 ng mL^−1^ and 750 ng mL^−1^) for 6 and 24 hours or with 1μM CL316.243 (Cayman Chemicals) for 24 hours for acute response. Cells were treated with S100A4 (150 and 750 ng mL^−1^) during all the differentiation process for chronic response.

#### 2.4.3 Gene expression

Visceral adipose tissue or cells were collected in liquid nitrogen. Total RNA was extracted using a column‐affinity–based methodology (NucleoSpin RNA II; Macherey‐Nagel, Duren, Germany). RNA yield was determined by spectrophotometry (NanoDrop). Five hundred nanograms of total RNA was retrotranscribed into cDNA using random hexamer primers and Multiscribe reverse transcriptase (TaqMan reverse transcription reagents, Thermo Fisher Scientific), following the manufacturer's instructions. Platinum Quantitative PCR SuperMix‐UDG with ROX reagent (Themo Fisher) was used as master mix reagent and expression levels of each gene of interest were assessed with the specific TaqMan probes (Thermo Fisher). mRNA levels of browning markers including uncoupling protein 1 (*UCP1*), peroxisome proliferator‐activated receptor gamma coactivator 1‐alpha (*PGC‐1α*) and inflammatory markers including monocyte chemoattractant protein‐1 (*MCP‐1/CCL2*), interleukin‐6 (*IL‐6*), or adipogenesis markers such as adiponectin (*ADIPOQ*) were assessed.

### 2.5 Statistical analysis

Unless otherwise stated, data are presented as mean ± SEM. Normality was tested using the Shapiro‐Wilk test. To analyse potential associations with other variables, standard least‐squares linear regression in JMP v14.1 was performed (SAS Institute Inc, Cary, NC) with log‐transformed circulating S100A4 levels as the independent variable, sex, age, and BMI (BMI‐SDS for children) as covariates, and either HOMA‐IR, insulin, HbA1c, or HMW‐adiponectin (HMW‐Adp) as the independent variable. Two‐tailed test, Mann‐Whitney *U* test for non‐normal distributed data, or one‐way ANOVA with Tukey's post hoc test for more than two groups were applied to determine statistical significance. *P* < .05 was considered significant for all analyses.

The Institutional Ethics Committee, in accordance with the Declaration of Helsinki, approved the study with adult subjects (CP15/00106 and PI17/01455). The children cohort study was approved by the Sant Joan de Déu Hospital's Ethic Committee (Comité Ètic d' Investigació Clínica—CEIC PIC‐21‐12).

## 3 RESULTS

### 3.1 Circulating S100A4 levels are elevated in both prepubertal children and adult subjects with obesity

Both prepubertal children and adults with obesity showed higher insulin resistance (estimated by HOMA‐IR) compared with their normal‐weight counterparts (Table 1). However, glucose and HbA1c levels in children with obesity were not altered compared with their normal‐weight controls, while they were significantly elevated in adults with obesity (Table 1). Both children and adults with obesity showed higher triglyceride levels, lower HDL‐cholesterol, and lower HMW‐Adp concentration than normal‐weight subjects (Table 1). Circulating S100A4 concentration was measured and significantly elevated levels in both prepubertal children (1.5‐fold, *P* = .002) and adult subjects with obesity (2.2‐fold, *P* < .001, Table 1) were found compared with normal‐weight controls.

**Table 1:** Anthropometric and metabolic parameters

Children	Normal weight(n = 15)	Obesity (n = 50)	*P* Value
Age, y	8.4 (0.8)	9.0 (1.1)	.060
Sex (F/M)	7/8	28/22	n.a.
BMI‐SDS	0.04 (0.86)	3.41 (0.75)	**<.001**
Glucose, mg dL^−1^	86 (6)	85 (8)	.620
Insulin, m.u.int/L	3.1 (1.6)	13.4 (6.6)	**<.001**
HbA1c, %	5.3 (0.2)	5.3 (0.2)	.891
HOMA‐IR	0.67 (0.36)	2.82 (1.44)	**<.001**
Triglyceride,^a^ mg dL^−1^	43 [15]	70 [36]	**<.001**
LDL‐Cho, mg dL^−1^	91 (19)	103 (23)	.054
HDL‐Cho mg dL^−1^	62 (11)	44 (10)	**<.001**
Cholesterol mg dL^−1^	162 (21)	164 (24)	.770
S100A4,^a^ ng mL^−1^	48 [28]	74 [39]	**.002**
HMW‐Adp,^a^ ng mL^−1^	5208 [3902]	3496 [2477]	**.006**
Adults	Normal weight (n = 16)	Obesity (n = 43)	
Age, y	45 (9)	46 (11)	0.933
Sex (F/M)	13/3	32/11	n.a.
BMI, kg m^−2^	24.2 (2.3)	45.7 (7.4)	**<.001**
Glucose, mg dL^−1^	89 (8)	109 (29)	**.002**
Insulin, m.u.int/L	6.9 (4.9)	14.0 (15.6)	**.008**
HbA1c, %	5.2 (0.2)	5.8 (0.8)	**<.001**
HOMA‐IR	1.6 (1.3)	4.0 (5.2)	**.003**
Triglyceride,^a^ mg dL^−1^	62 [20]	127 [53]	**<.001**
LDL‐Cho, mg dL^−1^	103 (23)	98 (34)	.516
HDL‐Cho, mg dL^−1^	67 (15)	42 (9)	**<.001**
Cholesterol, mg dL^−1^	188 (30)	164 (38)	**.021**
S100A4,^a^ ng mL^−1^	52.5 [33.8]	114.8 [86.6]	**<.001**
HMW‐Adp,^a^ ng mL^−1^	5917 [4651]	3042 [1984]	**.008**

*Note*. Data for normally distributed variables are shown as mean (SD), and two‐tail Student's *t* test was applied to compare groups.

Abbreviations: BMI‐SDS, standardized body mass index; HbA1c, glycated haemoglobin; HDL‐Cho, high‐density lipoprotein; HMW‐Adp, high molecular weight adiponectin; HOMA‐IR, homeostatic model assessment‐insulin resistance; LDL‐Cho, low‐density lipoprotein.

a Non‐normally distributed variables are shown as median [IQR], and groups were compared using the two‐tail Mann‐Whitney *U* test. Bold font indicates *P* < .05.

### 3.2 S100A4 levels are associated with HOMA‐IR in adults but not in prepubertal children

**Figure 1:**
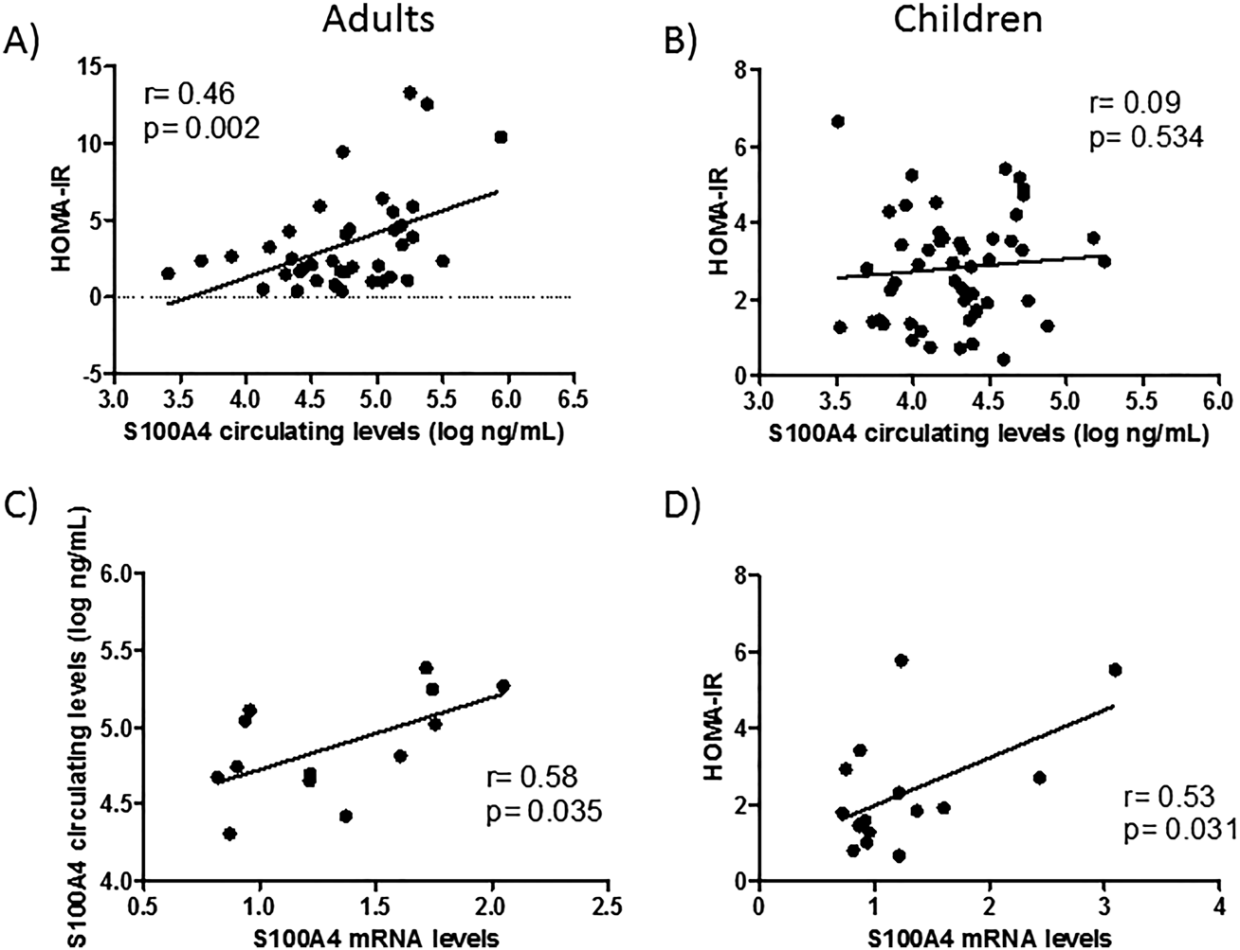
S100A4 is expressed in visceral white adipose tissue (vWAT) and correlates with circulating levels and homeostatic model assessment‐insulin resistance (HOMA‐IR). (A‐B) Bivariate correlation plots between log‐transformed circulating S100A4 levels and HOMA‐IR in (A) adults and (B) children with obesity. (C‐D) Bivariate plot between *S100A4* mRNA in vWAT and (C) circulating levels and (D) HOMA‐IR. *S100A4* gene expression is expressed as arbitrary units of *S100A4* mRNA levels relative to the expression of the housekeeping transcript *PPIA*. ^*^ *P* < .05

Then the association between circulating S100A4 levels and insulin resistance in the two different cohorts was tested. Given that obesity has a major impact on both S100A4 levels and insulin resistance, the focus of the analysis was on subjects with obesity to avoid this confounding factor. Circulating S100A4 levels were strongly correlated to HOMA‐IR in adult subjects with obesity (Figure 1A) and were maintained after adjusting for sex, age, and BMI (*βstd* = .42, *P* = .008, Table 2). However, no correlation between S100A4 and HOMA‐IR was found in prepubertal children with obesity (Figure 1B) before or after adjusting for sex, age, and BMI‐SDS (*βstd* = .12, *P* = .356, Table 2). A modest trend towards a correlation with HMW‐Adp was observed in children, without reaching statistical significance (*βstd* = .27, *P* = .061, Table 2).

**Table 2:** Correlations between circulating S100A4 levels and physiologic variables

S100A4	Children		Adults	
Variables	*βstd*	*P* value	*βstd*	*P* value
HOMA‐IR	.12	.356	**.42**	**.008**
Insulin	.06	.650	**.43**	**.008**
HbA1c	.13	.391	.25	.106
HMW‐Adp	.27	.061	.16	.445

### 3.3 S100A4 gene expression in WAT is associated with S100A4 circulating levels and HOMA‐IR in adult subjects with obesity

To evaluate the potential role of WAT in S100A4 secretion, S100A4 mRNA levels were measured in visceral WAT (vWAT) samples from adult patients with obesity. S100A4 expression in this tissue showed a positive correlation with its circulating levels and HOMA‐IR (Figure 1C,D).

### 3.4 S100A4 exerts anti‐inflammatory effects in vascular smooth‐muscle cells in vitro

**Figure 2:**
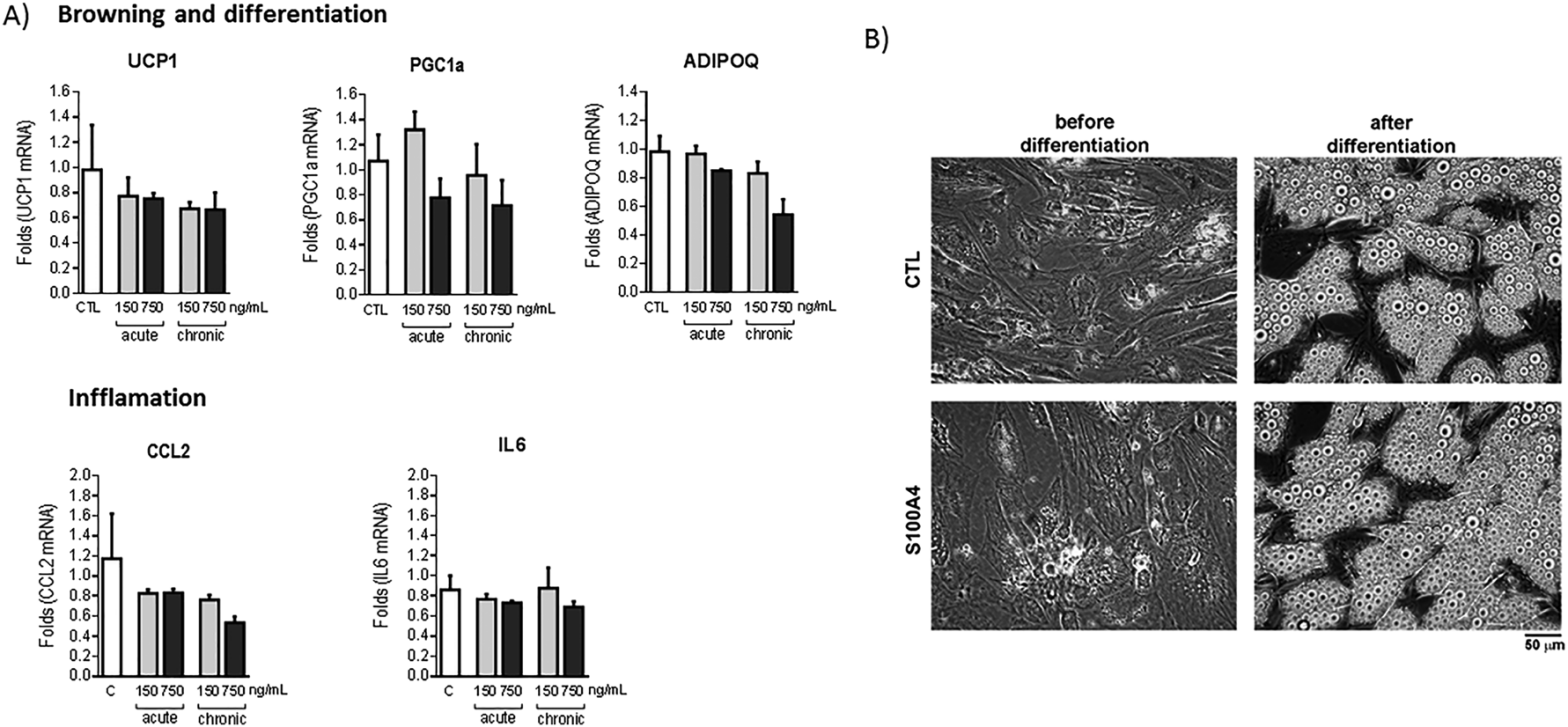
Treatment with S100A4 does not cause changes in human adipocytes. (A) Relative mRNA levels of different markers of adipogenesis, thermogenic capacity, and inflammation in human adipocytes treated with 150 or 750 ng mL^−1^ S100A4 for 24 hours or during differentiation, where indicated. Expression was normalized to *18S*. (B) Representative optical microscopy images from beige adipocytes treated with 750 ng mL^−1^ S100A4 since day 1 of differentiation. (^*^ *P* < .05) relative to control. The bars represent means ± SEM

**Figure 3:**
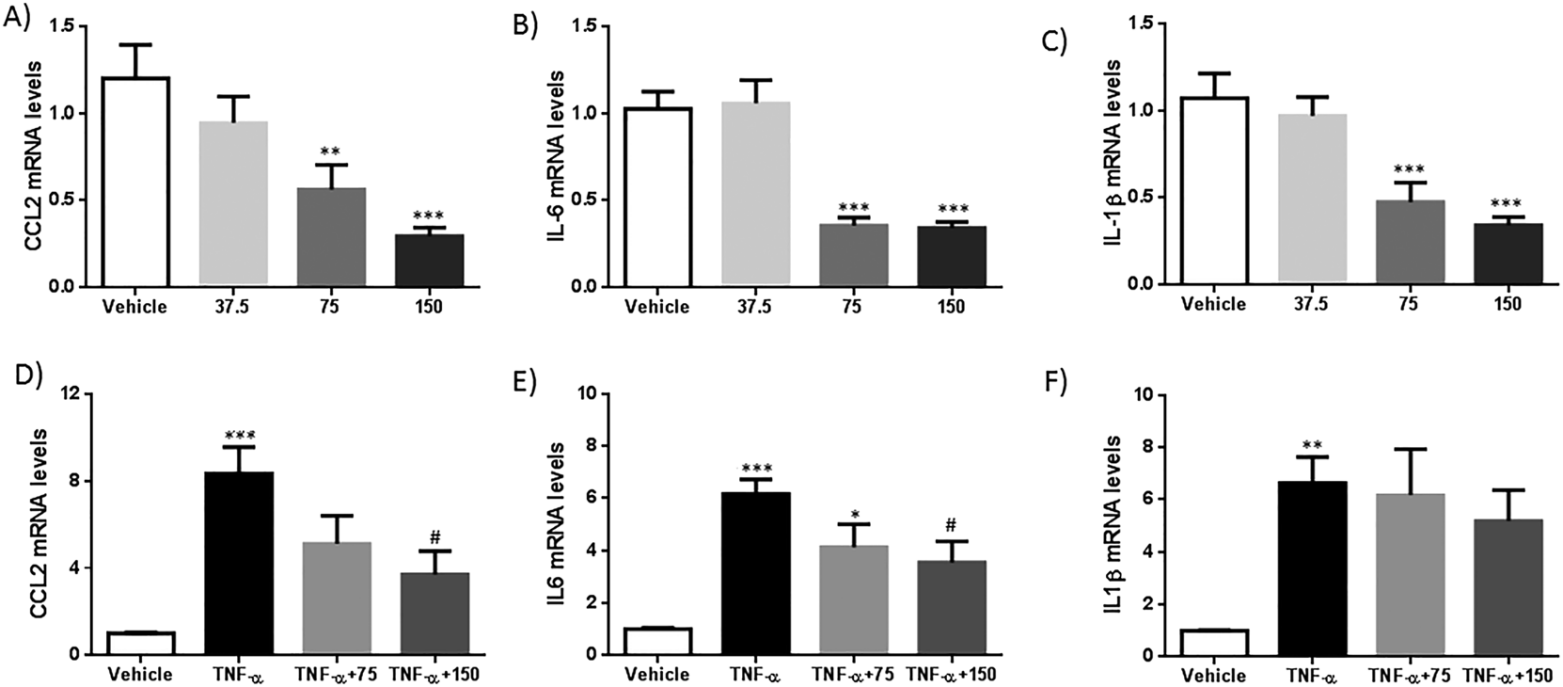
Treatment with S100A4 reduces the expression of pro‐inflammatory marker genes in human vascular smooth muscle cell (VSMC). Human VSMCs were exposed to S100A4 (37.5, 75, or 150 ng mL^−1^) or/and TNF‐α for 24 h. (A‐C) Expression of *CCL2*, *IL6*, and *IL1β* assessed by real‐time PCR and normalized to *GAPDH* 24 h after incubation with S100A4 (37.5, 75, or 150 ng mL^−1^). (D‐F) Expression of *CCL2*, *IL6*, and *IL1β* assessed by real‐time PCR and normalized to *GAPDH* 24 h after incubation with TNF‐α or TNF‐α + S100A4 (75 or 150 ng mL^−1^). Values are shown as mean ± SEM (n = 5); one‐way ANOVA was implemented to compare different treatments (^***^ *P* < .001, ^**^ *P* < .01, and ^*^ *P* < .05 vs vehicle; ^#^ *P* < .05 vs TNF‐α)

Since WAT seems to play a role in the secretion and function of S100A4, the aim was to evaluate the effects of this adipokine in two different cell types with a relevant role in adipose tissue expandability during obesity: adipocytes and VSMC. For these experiments, human adipocytes and VSMC were incubated with S100A4 for 24 hours (acute) or during 1 week of differentiation for human adipocytes (chronic). No effects were found in *UCP1*, *PGC‐1α*, *CCL2*, *IL‐6*, or *ADIPOQ* in human adipocytes (Figure 2). In human VSMC, a dose‐response curve with 37.5, 75, and 150 ng mL^−1^ of S100A4 for 24 hours was performed, showing that only the incubation with 75 and 150 ng mL^−1^ significantly decreased *CCl2*, *IL‐6*, and *IL‐1β* mRNA levels. No effect was observed with the lowest dose of S100A4 (37.5 ng mL^−1^). Furthermore, only the dose of 150 ng mL^−1^ partially blocked the increase of *CCl2* and *IL‐6* (but not *IL‐1β*) expression induced by incubation with TNF‐α, used as a positive control for inflammation (Figure 3).

## 4 DISCUSSION

To the best of our knowledge, this is the first time that S100A4 circulating levels are reported to be elevated in prepubertal children with obesity compared with normal weight healthy controls. As expected from previously published data (ref Arner), S100A4 levels were also elevated in our cohort of adult patients with severe obesity compared with normal‐weight controls and showed a correlation with insulin resistance. Surprisingly, the association between S100A4 and insulin resistance was not present in children.

S100A4 levels have been reported to be increased in human adults with obesity, being positively associated with sWAT dysfunction, inflammation, and insulin resistance.^17^ Other researchers have shown that S100A4 deficient mice were more susceptible to develop obesity and insulin resistance after a high‐fat diet, suggesting a protective role for S100A4 in diet‐induced obesity.^18^ Furthermore, overexpressed S100A4 inhibited adipogenesis and decreased inflammatory markers in 3T3‐L1 adipocytes.^18^ Our results suggest a potential explanation for the controversial role of S100A4 in obesity and insulin resistance. Our cohort of children with obesity showed hyperinsulinaemia, but this excess of insulin secretion was able to maintain normal glucose levels. Researchers have reported an induction of adiponectin by S100A4 in 3T3‐L1 adipocytes and in mice.^18^ Although there was no correlation between S100A4 and HMW‐Adp, it is plausible that the elevated S100A4 levels may play a beneficial role during early states of obesity in an attempt to balance the hyperinsulinaemia and the potential development of insulin resistance in this population in which obesity is only at the beginning of eliciting metabolic dysfunction.

Moreover, our in vitro experiments demonstrate a beneficial direct effect of S100A4 on VSMC by reducing inflammatory markers. S100A4 is secreted by adipocyte precursors and immune cells^17^ and, during obesity, both an alteration of adipogenesis and the recruitment of immune cells occur (reviewed in^24^). In addition, new blood vessel formation (angiogenesis), including VSMC, is required for a correct adipose tissue expandability.^25^ Moreover, inflammation and vascular dysfunction contribute to the development of obesity and insulin resistance.^26^ Therefore, it is tempting to speculate that S100A4 secretion by immune cells infiltrated in adipose tissue during obesity acts on VSMC and thereby regulates the vascular tone, distribution of blood flow, as well as angiogenesis, inflammatory processes, and redox status. It is plausible that that this beneficial effect could contribute to improving the inflammatory state during the early stages of obesity development, delaying the development of adipose tissue dysfunction and insulin resistance.

In accordance with other researchers,^17^ no direct effect of S100A4 on adipocytes was observed possibly due to the lack of specific receptors, suggesting that these cells are not a target for S100A4 action. In agreement with Arner et al,^17^ who postulated that S100A4 is an adipokine, our data showed that S100A4 is expressed in WAT. In addition, S100A4 vWAT gene expression correlated with circulating levels, suggesting that vWAT is a source of circulating S100A4. Moreover, S100A4 mRNA and circulating levels positively correlated with glucose homeostasis parameters, suggesting a role of this adipokine in the metabolic status of these patients. An intriguing possibility is that a chronic release of this molecule in the obesity status could lead to an S100A4 resistant situation, in a similar way to what has been shown for other molecules including leptin and FGF21.^27^, ^28^ Further studies are warranted to determine the occurrence of an S100A4 resistant state in obesity.

The limitations of this study include the lack of adipose tissue samples in children to analyse the molecular mechanisms involved in the elevation of S100A4 and the lack of correlation with insulin resistance. Moreover, HOMA‐IR was used as a marker of insulin resistance due to the lack of a clamp assay. Another important limitation is that our vascular cellular model comes from aorta and not from adipose tissue vessels. In addition, longer and chronic S100A4 exposure would be desirable to evaluate the long‐term effect of S100A4 in vitro. The strengths include the large cohort of plasma samples from prepubertal children with obesity and the availability of additional physiologic parameters allowing to identify the associations between S100A4 and other surrogates of metabolic state.

In conclusion, our human data demonstrate for the first time that S100A4 circulating levels are higher in children with obesity compared with normal‐weight individuals but do not correlate with insulin resistance as in adult subjects. Moreover, our in vitro results suggest a potential anti‐inflammatory effect of S100A4 in VSMC. Further studies will be necessary to determine whether S100A4 can be a therapeutic target for obesity and the development of insulin resistance associated with this condition.

## AUTHOR CONTRIBUTIONS

Siri D. Taxerås, María Galán, and Laura Campderros researched and analysed the data, contributed to data interpretation, and wrote the manuscript. Irene Piquer‐Garcia, Silvia Pellitero, Eva Martínez, Rocío Puig, and Icíar Lucena researched and analysed the data. Jordi Tarascó, Pau Moreno, and José Balibrea obtained adipose tissue samples and contributed to data analysis and interpretation. Joan Bel, Marta Murillo, and María Martínez contributed to study design and reviewed/edited the manuscript. Marta Ramon‐Krauel and Manel Puig‐Domingo contributed to study design and data interpretation. Francesc Villarroya, Carles Lerin, and David Sánchez‐Infantes contributed to study design, researched data, and wrote/reviewed/edited the manuscript. Each author listed in the manuscript has seen and approved the submission of this version of the manuscript and takes full responsibility for the contents.

## CONFLICT OF INTEREST

The authors declared no conflict of interest.

## FUNDING INFORMATION

D. S. I. and M. G. are investigators of the Miguel Servet Fund from Carlos III National Institute of Health, Spain. This study was supported by Instituto de Salud Carlos III, by the Fondo Europeo de Desarrollo Regional (FEDER), Madrid, Spain (CP15/00106 and FIS17/01455), and by K‐sted 651010‐project number 70084900‐analyse number M50676 from Norwegian University of Science and Technology (NTNU).
